# Seaweed Loads Cause Stronger Bacterial Community Shifts in Coastal Lagoon Sediments Than Nutrient Loads

**DOI:** 10.3389/fmicb.2018.03283

**Published:** 2019-01-09

**Authors:** Tânia Aires, Gerard Muyzer, Ester A. Serrão, Aschwin H. Engelen

**Affiliations:** ^1^Centro de Ciências do Mar (CCMAR), Centro de Investigação Marinha e Ambiental (CIMAR), Universidade do Algarve, Faro, Portugal; ^2^Microbial Systems Ecology, Department of Freshwater and Marine Ecology, Institute for Biodiversity and Ecosystem Dynamics, University of Amsterdam, Amsterdam, Netherlands

**Keywords:** marine sediments, microbiome, 16S amplicon sequencing, eutrophication, algal bloom

## Abstract

The input of nutrients from anthropogenic sources is the leading cause of coastal eutrophication and is usually coupled with algal/seaweed blooms. Effects may be magnified in semi-enclosed systems, such as highly productive coastal lagoon ecosystems. Eutrophication and seaweed blooms can lead to ecosystem disruption. Previous studies have considered only one of these factors, disregarding possible interactive effects and the effect of the blooming species’ identity on sediment bacterial communities. We tested the effect of experimental nutrient loading and two common blooming seaweeds (*Ulva rigida* and *Gracilaria vermiculophylla*) in coastal lagoon sediments, on the structure of bacterial communities (using 16S rRNA amplicon sequencing) and corresponding putative functional potential (using PiCRUSt). At the Operational Taxonomic Unit (OTU) level, the addition of nutrients reduced bacterial community α-diversity and decreased the abundance of sulfate reducers (*Desulfobacterales*) compared to sulfur oxidizers/denitrifiers (*Chromatiales* and *Campylobacterales*), whereas this was not the case at the order level. Seaweed addition did not change bacterial α-diversity and the effect on community structure depended on the taxonomic level considered. The addition of *Gracilaria* increased the abundance of orders and OTUs involved in sulfate reduction and organic matter decomposition (*Desulfobacterales, Bacteroidales*, and *Clostridiales*, respectively), an effect which was also detected when only *Ulva* was added. Nutrients and the seaweeds combined only interacted for *Ulva* and nutrients, which increased known sulfide oxidizers and denitrifiers (order *Campylobacterales*). Seaweed enrichment affected putative functional profiles; a stronger increase of sulfur cycling KEGG pathways was assigned to nutrient-disturbed sediments, particularly with the seaweeds and especially *Ulva*. In contrast, nitrogen and sulfur cycle pathways showed a higher abundance of genes related to dissimilatory nitrate reduction to ammonium (DNRA) in *Ulva*+nutrients treatments. However, the other seaweed treatments increased the nitrogen fixation genes. Thiosulfate reduction, performed by sulfate-reducing bacteria, increased in seaweed treatments except when *Ulva* was combined with nutrients. In conclusion, the *in situ* addition of nutrients and the seaweeds to intertidal sediments affected the bacterial communities differently and independently. The predicted functional profile suggests a shift in relative abundances of putative pathways for nitrogen and sulfur cycles, in line with the taxonomic changes of the bacterial communities.

## Introduction

Coastal systems are affected by multiple anthropogenic stressors of both marine and terrestrial origin (Tallis et al., 2008), such as eutrophication. One of its main causes is the high input of phosphorus (P) and, more importantly, nitrogen (N) from agricultural fertilizer runoff and wastewater discharge (Nixon, 1995). This will, consequently, lead to elevated atmospheric N concentrations and increased atmospheric deposition of N (Holland et al., 2005), resulting in a dramatic rise of the amount of bioavailable N in natural habitats (Galloway and Cowling, 2002). In coastal marine systems, N is often a limiting nutrient (Taylor et al., 1995), but excessive nutrient inputs drive microbial communities to create biogeochemical feedbacks that promote the availability of N and P (Howarth et al., 2011). This positive feedback stimulates eutrophication and has negative effects, such as harmful or opportunistic blooms of seaweed (hereafter also referred as alga/algae) and the outgrowth of phytoplankton and epiphytic algae (Hauxwell et al., 2001). These blooms are the primary cause for oxygen depletion, which leads to hypoxia/anoxia with consequences for biodiversity (Isbell et al., 2013; Leff et al., 2015) and society (Leliaert et al., 2009).

Following seaweed-blooms, the decay of algal biomass increases oxygen consumption and results in anoxic conditions (Meyer-Reil and Köster, 2000). This, in turn, leads to a disruption in the ecosystem function and sediment biogeochemical cycling (Hale et al., 2016). Besides the “eutrophication-dependent” blooming seaweed, invasive algae may also proliferate due to a lack of grazing in the invaded areas (Engelen et al., 2011), which will later result in, mostly drifting, blooms that can lead to similar ecosystem disruption.

Bacteria play a determinant role in the biogeochemical cycles (Brune et al., 2000; Spring et al., 2000) of sulfur, nitrogen, and carbon, among others (Wetzel, 2000; Borsodi et al., 2003; Micsinai et al., 2003), and are responsible for nutrient transformation and organic matter decomposition (Zheng et al., 2014). Therefore, bacteria are crucial players in many ecosystem processes, such as the conversion of organic and inorganic compounds and, most importantly, the removal of anthropogenic nitrogen in coastal lagoons before its entry into the coastal ocean (Brin et al., 2010). Besides assimilation through sediments and into biological biomass and anammox, an essential process for nitrate removal is respiratory denitrification (Thamdrup, 2012). In contrast, dissimilatory nitrate reduction to ammonium (DNRA) retains nitrogen but in a more biologically available form (Tiedje, 1988). These two competing processes are mediated by bacteria and are dependent on the C/NO_3_^-^ ratio (Burgin and Hamilton, 2007). Hence, identifying bacterial community shifts and their driving forces under common anthropogenic stresses is an essential step in predicting ecosystem responses and health during eutrophication and associated algal-bloom episodes.

The effects of eutrophication on biogeochemical cycles in sediments, at the chemical level, have been described by measuring the transformation rates of each final product (e.g., Sanz-Lázaro et al., 2015; Spivak and Ossolinski, 2016). Microbial processes have long been described and correlated with the degree of eutrophication in lakes (Chróst and Siuda, 2006). However, only in the last decade, advances in molecular techniques and the “omics” field allowed the expansion of our knowledge on the anthropogenic effects on coastal sediments through the study of their bacterial communities. For example, different levels of undifferentiated organic pollution revealed a consistent shift in microbial communities (Sinkko et al., 2013; Xiong et al., 2014), with bacterial taxa shifting to a Deltaproteobacteria-dominated guild, from which sulfate reducers prevailed. Other studies have reported contradictory results where sediment bacterial communities demonstrate resistance to nutrient inputs (Bowen et al., 2011), possibly by inducing dormancy in part of the community, which decreases diversity just on the active bacteria of the total community (Kearns et al., 2016). Hence, there are still large uncertainties regarding the prediction of the effects of eutrophication on sediment bacterial communities.

Algal blooms and bacterial communities are bidirectionally linked. Besides the pivotal role of bacteria in main biogeochemical processes and nutrient cycling, there is also evidence that they can regulate algal blooms (Kodama et al., 2006). Some planktonic bacterial groups can act either as growth stimulators (Anderson et al., 2012) or control factors. An example are the Cytophaga-Flavobacterium-Bacteroides (CFB), usually linked to the degradation of high molecular weight dissolved organic matter in the final stages of the blooms (Ishida et al., 1997; Jones et al., 2010). Consequently, bacterial communities are likely to change during a bloom (Grossart et al., 2005; Rooney-Varga et al., 2005; Chen et al., 2015), which makes it essential to understand how certain bacterial communities interact with algal blooms and determine the stage of the bloom at which this takes place. To date, most studies focused on the communities present in the water column or directly associated with the blooming seaweeds. However, earlier studies showed an increase of sulfur pools in eelgrass sediments by algal mats (Holmer and Nielsen, 2007), which might also be reflected in the sediment microbial community. Recently, Morrison and colleagues (2017) showed that bacterial communities from seaweed-enriched sediments, sourced from different inoculums, differed from this pre-enrichment community. Most of their distinctions were more inoculum-source than seaweed-species related but, in all of the treatments, the shifts were mainly toward the increase of bacterial lineages that are related to organic matter degradation. This suggests that algal blooms would lead to bacterial communities dominated by organic matter degraders and that algal identity is not of great importance.

Although nutrient inputs (which potentially affect sediment bacterial communities) are usually coupled with seaweed blooms (which itself triggers a shift in the bacterial community), their combined effects on sediment bacterial communities are still poorly understood. An important role of this interaction is, however, suggested by the observation that the composition of sediment bacterial communities only changed in response to nutrient fertilization in an area colonized by filamentous algae; possibly due to a shift in the carbon supply from those algae (Bowen et al., 2009). Moreover, changes in the bacterial community of both nutrient-poor and nutrient-rich (N and P naturally released by dreissenid mussels) freshwater sediments were shown to be associated with the presence of benthic algae, where bacterial taxa capable of degrading algal cellulose increased (Lee et al., 2015). Besides these few sources of evidence on the importance of nutrient and algal interactions for sediment bacterial communities, this topic strongly lacks further empirical and experimental evidence.

In this study, we conducted the first experimental field test of how nutrient input and algal loading (separately and combined) affect the sediment bacterial community structure in a coastal lagoon, and we predicted hypothetical functional consequences based on the resulting data. We used macroalgae (seaweeds) with different blooming strategies: *Ulva rigida*, whose blooms are driven by eutrophication (Fletcher, 1996; Gao et al., 2016), and *Gracilaria vermiculophylla*, which is invasive in the studied area, lacks natural predators, and often experiences blooms (Thomsen and McGlathery, 2007). These two seaweeds also present different structural profiles, with *G. vermiculophylla* showing a more ligneous/fibrous structure, highly resistant to anaerobic conditions and desiccation, in contrast with *U. rigida*, which has a thinner/fragile blade prone to desiccation, having a faster turnover in similar conditions. The distinct evolutionary histories of these two seaweeds are also noteworthy as they resulted in differentiated bacterial communities (Lachnit et al., 2011). We analyzed the bacterial community structure/composition using 16S rRNA gene amplicon sequencing on an Illumina MiSeq system in order to test three main hypotheses: (i) sediment bacterial community diversity will decrease and bacteria associated with nitrogen and sulfur cycles will change composition to respond to nutrient enrichment, (ii) seaweed enrichment will also affect bacteria related to nitrogen and sulfur cycles and decomposition of organic matter (carbon cycling), and (iii) the shifts in bacterial community will be cumulative (more evident) when the sediment is loaded with both nutrients and seaweed. By using two different seaweed species with different blooming strategies, we also aim to test the generality of the seaweed effects on bacterial communities. Last, we assess the predicted functional profiles and relate these to the main shifts in bacterial groups, linking them to possible responses to such environmental disturbances. This will contribute to a better understanding of the impact of eutrophication on its initial (nutrient increase) and secondary (algal bloom) stages, and the differentiated impact of different blooming algal species. This first experimental field test of effects of both nutrients and algal blooms reveals their individual and combined differentiated effects on bacterial communities in intertidal sediments.

## Materials and Methods

### Study Site

The field experiment was performed during the summer of 2011 in the Ria Formosa lagoon, a warm temperate mesotidal system, located on the Atlantic coast of southern Portugal (37°N, 8°W). The lagoon is characterized by salt marshes, mudflats, and channels. It extends along the coast for about 55 km and is 6 km wide, with several connections to the Atlantic Ocean, ensuring water replacement during tidal cycles (1.5–3.0 m). Freshwater inflow is low and the salinity remains close to 36 (Machás et al., 2006). The experiment was conducted on a tidal flat with a vertical intertidal gradient of about 2 m. On average, *Zostera noltii* cover was 77%, with ∼8000 shoots/m^2^; 85 mm leaf length and above ground 219 gDW/m^2^, with a low abundance of seaweeds (19 gDW/m^2^) (DW – dry weight). Ambient nutrient levels at the experimental site were 1.0 μM NH_3_ (Ammonia), 0.6 μM PO_4_ (Phosphate), 1.0 DIN (dissolved inorganic nitrogen), and the average sediment organic matter was 3.6%DW during the experimental period. Seawater daily average temperature was 24.5°C (obtained from two HOBO loggers), and salinity stood at 36 psu, for the duration of the experiment.

### Field Experiment and Experimental Design

**FIGURE 1:**
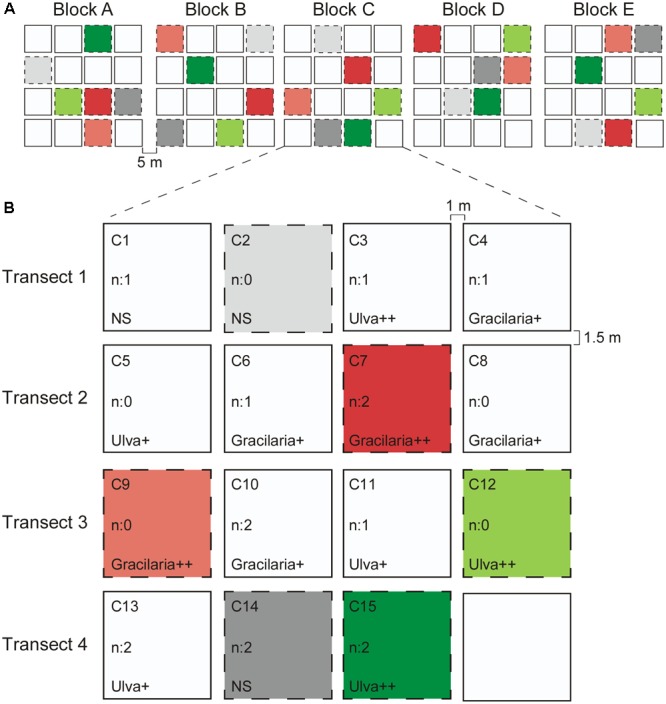
Experimental design. **(A)**. Five blocks along a transect with 5 m in between the blocks. **(B)** Example of one of the blocks showing all the different treatment plots. Between two plots within a transect there is a distance of 1 m and the distance between two plots of adjacent transects is 1.5 m. The different treatments considered in this study are colored and are as follow: Light gray: *n*: 0 = no nutrients, NS = no seaweeds; Dark gray: *n*: 2 = 250 g N m^-2^ of nutrients added; Light red: *n*: 0 = no nutrients, Gracilaria++ = 3000 g WW m^-2^ of *Gracilaria* added; Dark red: *n*: 2 = 250 g N m^-2^ of nutrients added, Gracilaria++ = 3000 g WW m^-2^ of *Gracilaria* added; Light green: *n*: 0 = no nutrients, Ulva++ = 3000 g WW of *Ulva* m^-2^ added; Dark green: *n*: 2 = 250 g N m^-2^ of nutrients added, Ulva++ = 3000 g WW of *Ulva* m^-2^ added. Each block was composed by the same treatments and each one was sampled for each treatment totalling five replicates/treatment. No colored plots were not considered in this study.

Nutrient levels and seaweed abundances were manipulated in treatment plots of 0.25 m^2^, marked with zinc wire quadrats fixed on the sediment by two (about 30 cm long) zinc wire pegs in opposite corners, entirely open for natural intertidal conditions at the site. Quadrats were separate from each other by 1–1.5 m in blocks of 15 (1 per treatment), distanced by 5 m and set up on June 6th, 2011 (see Figure 1).

The effect of eutrophication was tested with nutrient treatments consisting of (i) no nutrient (reference – N0) or (ii) 250 g slow diffusing N m^-2^ addition (N2). The nutrient addition was accomplished with a slow-release granulated fertilizer (Basacote Plus 3M, with 16% N, 8% P, and 12% K), which at the daily average seawater temperature during the experiment would release for about 3 months. To test the effects of the seaweeds, either (i) no seaweed (reference – SW0) or (ii) 3000 g m^-2^ (wet weight) of seaweed was added (for both species – Gracilaria, Ulva). In the Ria Formosa lagoon, both *U. rigida* and *G. vermiculophylla* are locally abundant; therefore, we used both seaweed species in the seaweed-enrichment treatment to test for species-specific effects. Overall, combinational treatments of two nutrient level treatments and two seaweed abundance treatments with two different seaweed species (*Ulva* and *Gracilaria*) were applied, and the positions of the treatment plots in a block were randomized. The seaweed biomass utilized was in accordance to biomasses found in the field (Cardoso et al., 2004) and used by others (Cardoso et al., 2004; Teichberg et al., 2010). The nutrient and seaweeds were added during low tide on June 13th, 2011.

The seaweeds (*Ulva* and *Gracilaria*) were collected in Quinta do Lago (Ria Formosa) 3 weeks before the start of the experiment and preserved in flow-through tanks at the CCMAR marine station. Before adding the algal biomass to the field, the seaweeds were cleaned of associated fauna. Treatments were randomly assigned to plots within blocks. Fertilizer granules were placed into the top centimeters of sediment and homogeneously distributed on the plot. If a plot had to be filled with both nutrients and seaweeds, the nutrients were put in first and, subsequently, the seaweeds were attached. Seaweed biomass was secured in plots by using five pegs per plot. Prior to the experiment, all plots were cleared of seaweed and all the plots received five pegs (including the no seaweed reference plot).

The experiment ran for 5 weeks (13th of June till 20th of July) during which photos of each plot were taken on the 17th and 30th of June and the 8th and 11th of July, to check whether the coated slow-releasing fertilizer granules and seaweeds were still in position. No other seaweed species than those added were observed in any of the plots during any of the surveys. The duration was chosen based on the commonly used time in drift algal-seagrass studies and reflects the short persistence time of drift algae in individual plots (Cardoso et al., 2004; Thomsen, 2010). By the end of the experiment, one replicate of 4 ml of the top few centimeters of sediment from each treatment was taken in cryovials from the correspondent plot in each of the five blocks (Figure 1) (totalling five replicates per treatment), flash frozen, and stored at -80°C until further processing. Seagrass root fragments (3.62 gDW ± 0.34 SE) were collected from each plot and these were processed for tissue nitrogen content as a proxy for plot-level nutrient status (Burkholder et al., 2007; Reynolds et al., 2018) using a Vario EL III elemental analyser (Elementar).

### DNA Extraction and Bacterial Tag-Encoded 16S Amplicon Sequencing

DNA was extracted with the PowerSoil^®^ DNA Isolation Kit (MO BIO, Laboratories, Inc.) following the MO BIO Vortex Adapter protocol. The 16S rRNA gene V4 variable region was amplified by PCR using the primers 515F/806R (Caporaso et al., 2012) and processed for MiSeq Illumina sequencing by Research and Testing laboratories (Lubbock, TX, United States)^1^. Samples were amplified for sequencing using a forward and reverse fusion primer. The forward primer was constructed with the Illumina i5 adapter (5′-AATGATACGGCGACCACCGAGATCTACAC-3′), an eight bp barcode, a primer pad, and the 515F primer (GTGCCAGCMGCCGCGGTAA). The reverse fusion primer was constructed with the Illumina i7 adapter (5′-CAAGCAGAAGACGGCATACGAGAT-3′), an eight bp barcode, a primer pad, and the 806R primer (GGACTACHVGGGTWTCTAAT). Primer pads were designed to ensure the primer pad/primer combination had a melting temperature of 63–66°C, according to methods developed by the lab of Patrick Schloss^2^ (Kozich et al., 2013). Amplifications were performed in two independent 25 μl PCR reactions (which were pooled for the final assay) with Qiagen HotStar Taq master mix (Qiagen Inc., Valencia, CA, United States), 1 μl of each 5 μM primer, and 1 μl of template. Reactions were performed on ABI Veriti thermocyclers (Applied Biosystems, Carlsbad, CA, United States) under the following thermal profile: 95°C for 5 min, then 35 cycles of 94°C for 30 s, 54°C for 40 s, 72°C for 1 min, followed by one cycle of 72°C for 10 min. Samples were pooled together in equal proportions, based on their molecular weight and DNA concentrations. Calibrated Ampure XP beads were used to purify samples. DNA libraries were prepared by using an Illumina TruSeq DNA library preparation protocol.

### Bacterial Community Characterization

#### 16S Sequence Analysis

Generated sequences were trimmed for quality and screened for a minimum read length of around 300 bp, sequences with ambiguous base calls were removed. Analyses were performed using the QIIME 1.8.0: Quantitative Insights Into Microbial Ecology (Caporaso et al., 2010) pipeline. Selected high-quality sequences were clustered into Operational Taxonomic Units (OTUs) within reads using the denovo OTU picking method. Representative sequences for each OTU were selected using the “most-abundant” method and OTU sequence alignment was carried out using PyNAST and Greengenes database v.13.8 (Caporaso et al., 2010). Taxonomic assignments were conducted through the UCLUST method with a 97% confidence threshold. Each OTU was assigned to its closest-matching description of taxon in the Greengenes database v.13.8 of 16S rRNA sequences (McDonald et al., 2012), and sequences were putatively assigned to a described taxon with a minimum threshold of 0.001 (default value). Eukaryote sequences (i.e., chloroplasts and mitochondria), as well as unassigned sequences, were excluded from the OTU table during downstream analyses. Due to the high variation in the number of high-quality sequences obtained among replicates (10311–124402 sequences), in the quality controlled OTU table the sample with the lowest number of sequences contained 10,311 reads. Therefore, all the samples were rarefied to this number of sequences for further statistical analyses (the rarefied table resulted in 18,278 unique OTUs and 289,576 sequences).

#### Statistical Analysis

The statistical tests performed in this study were carried out using the rarefied data and considered significant at *P* < 0.05. Low abundance OTUs (singletons and doubletons) were removed from the dataset. The homogeneity of multivariate dispersions (based on the mean distance to group centroid for all groups within each factor) was tested using a resemblance-based permutation test (PERMDISP) (Anderson et al., 2006) before confirming differences in community structuring among sampling groups (β-Diversity) – by applying a permutational multivariate analysis of variance (PERMANOVA) using Bray–Curtis dissimilarity matrices from square-root transformed data.

For the β-diversity assessment, a multivariate analysis of microbial diversity using permutational analysis of variance (PERMANOVA) and a canonical analysis of principal coordinates (CAP) were performed. PERMANOVA tested for differences among samples with *a priori factors*: Different Treatments: Nutrients (N2) vs. No-nutrients (N0), Seaweeds (Ulva and Gracilaria) vs. No-seaweeds (SW0), and the interaction between both [Reference (N0SW0), N2SW0, N0Ulva, N0Gracilaria, N2Ulva, N2Gracilaria] and differences within seaweeds’ treatment [No seaweeds (SW0) vs. *Gracilaria/Ulva*, and *Gracilaria* vs. *Ulva*]. A canonical analysis of principal coordinates (CAP) tested the assignment/clustering of treatments interaction (Nutrients X Seaweeds) as an *a priori* factor. Discriminant vectors with a Pearson correlation >0.6 were considered for the characterization of the taxa discriminating the multivariate patterns. Since not all the OTUs were assigned to the same taxonomic level, only bacterial orders were considered in order to have a homogeneous characterization (at the same taxonomic level for each vector). SIMPER analyses were performed to determine which taxa contribute most to differences among groups. All analyses were performed using the software program PRIMER-E+PERMANOVA v.6 (Clarke and Warwick, 1994).

To investigate the shifts in the bacterial community at a higher taxonomic level and consistently present through the different replicates, we filtered the OTU table, at the order level, for OTUs that were present in at least 60% of the replicates. A bar chart was generated with those orders remaining after filtration and considering only the ones with a relative abundance of above 1.5%. The putative roles of the main OTUs/orders found for each of the treatments were inferred from the literature. A heat map was built, using STAMP (Parks et al., 2014), where the different treatments were clustered independently using Ward’s clustering method (with a 0.75 threshold) for the main orders’ relative abundances (as used for the bar chart above).

Estimates of α-diversity were based on evenly rarefied OTU level and most common orders abundance matrices and included the diversity and evenness indicator Shannon index and the richness and abundance Indexes Chao I and Observed OTUs, as calculated in QIIME. In order to maximize comparability with the analysis of β-diversity, treatment effects on α-diversity were examined using univariate PERMANOVA, based on Euclidean distances (as in Hartmann et al., 2015). Using the compare_alpha_diversity.py script on QIIME, boxplots were generated for a visualization of the α-diversity differences among groups.

#### Functional Profile Prediction From 16S Data

In order to predict metagenomes, closed-reference OTU picking was performed using the subsampled reads against the GreenGenes reference database (version GG 13.5) using the pick_closed_reference_otus.py script from QIIME (Caporaso et al., 2010). This was done separately from the bacterial community composition analysis because PiCRUSt requires an older version of GreenGenes, as well as the closed reference OTU picking method, which would limit a more comprehensive and broad diversity analysis. PiCRUSt (Langille et al., 2013) was used online in the Galaxy environment following the PiCRUSt workflow from Huttenhower’s Lab^3^. This included 16S copy number normalization, metagenomes/function prediction, and function categorizing into KEGG pathways. General statistical analyses were performed using PERMANOVA following the same procedure as for the 16S rRNA gene analysis of the bacterial communities in PRIMER-E+PERMANOVA v.6 (Clarke and Warwick, 1994).

Based on the most important biogeochemical cycles in coastal sediments (i.e., sulfur and nitrogen cycles), candidate functional categories (KEGG classification, pathway category Level 3) were chosen and analyzed in detail for each treatment group. These analyses were performed with the software program STAMP v. 2.1.3 (Parks et al., 2014) using ANOVA and the multiple groups’ boxplot visualization for the Nutrient and Seaweed combined treatments. When statistical differences were found (*p* < 0.05), *post hoc* Turkey–Kramer tests were performed to identify the source of the variation. Besides, from the predicted metagenomes table calculated in PiCRUSt, the KEGG orthologues implicated in the different pathways of the sulfur and nitrogen cycle (according to the KEGG pathway database) were selected. The selected genes were pooled by pathway and plotted to compare relative abundances for each treatment.

To confirm if the closed-reference OTU table presents the same trend for the main orders as the OTU table used for the bacterial community analysis, the procedure described above, for the bar chart construction, was applied to the closed-reference OTU table. That was used to strengthen the assumptions on putative functions made for the main OTUs/orders characterized by 16S barcoding (see above). The OTU table was then filtered by the orders that were more abundant (based on the bar chart graph) in each treatment group (Reference, N2SW0, N0Grac, N2Grac, N0Ulva, and N2Ulva). PiCRUSt analyses were then repeated to target sulfur metabolism (found to be the most shifting function) in these orders and to check if the functional profile followed the same pattern as the community profile. To investigate the carbon cycle, we directly selected the Level 3 KEGG pathways that were related to carbon utilization and present in the PiCRUSt list. The statistical analyses and visualization of the results were performed with STAMP (Parks et al., 2014).

## Results

### Effects of Eutrophication and Algal Blooms on Bacterial Community Structure and Composition

Our raw dataset consisted of a total of 2,896,310 sequences. After quality control, and removal of chimeras, chloroplast, unassigned sequences, and singletons, a total of 1,114,886 high-quality sequences were retained for analysis (accessible through MG-RAST)^4^, consisting of a total of 21,812 unique OTUs (Supplementary Table S1).

#### OTU Level Analyses

**FIGURE 2:**
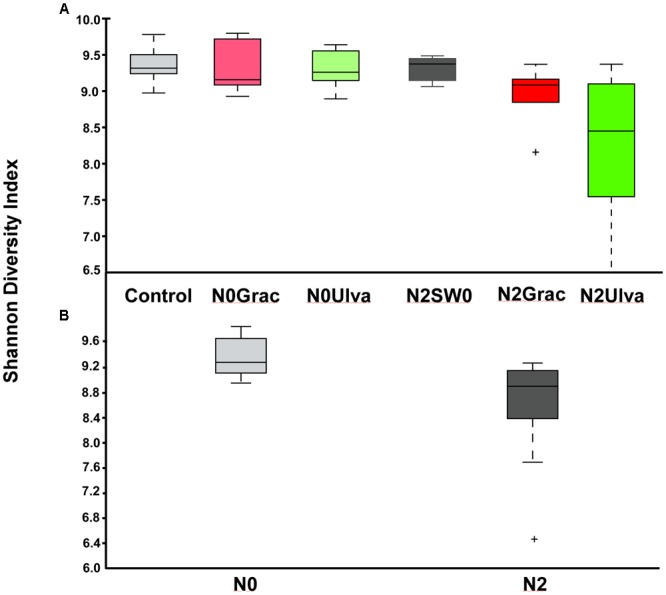
**(A)** Boxplot of the bacterial α-diversity using Shannon Diversity Index of intertidal sediment bacterial communities based on Illumina 16S rRNA amplicon sequencing for ambient conditions (Reference) and experimental conditions of nutrient (N) and seaweed (SW) addition. **(B)** Once significant differences were detected for nutrient addition, all non-nutrient vs. nutrient treatments were plotted together and new plots presented below the main plot. The line in the middle of each box is the median, crosses are outliers, and the whiskers represent error bars. From left to right: N0SW0 – No nutrients or seaweeds added (Reference), N0Grac – Only *Gracilaria* added, N0Ulva – Only *Ulva* added, N2SW0 – Only nutrients added, N2Grac – Both nutrients and *Gracilaria* added, N2Ulva – Both nutrients and *Ulva* added. Graphics were manipulated to both fit in the same figure. In addition, colors were changed so different treatment colors would match those of Figure 1.

Alpha diversity indices were calculated for each replicate of each treatment at both OTU and order levels (except for the Observed OTUs) and individual values are shown in Supplementary Table S2. Bacterial α-diversity was reduced by nutrient addition only when expressed by the Shannon index (Table 1 and Figure 2). Seaweed addition did not affect bacterial α-diversity according to any of the three indices used (Table 1).

**Table 1:** Results of PERMANOVA main test for the α-diversity indexes calculated: Shannon Diversity, ChaoI and Observed OTUs.

Source of variation	Pseudo-F (Shannon/ChaoI/Obs. OTUs)	P (*perm*) (Shannon/ChaoI/Obs. OTUs)	PERMDISP (P) (Shannon)
Nutrients	4.3878/5.8915E-2/0.46874	**0.044/**0.816/0.503	0.06
Seaweeds	2.4843/0.98266/1.4276	0.094/0.398/0.257	7
Nu × Se	1.938/0.35053/0.65214	0.157/0.716/0.512	8

*PERMANOVA values were calculated after Euclidian distances calculation for Shannon values. H_*0*_: there are no differences in the α-diversity for the different treatments, H_*0*_ rejected if: *p* < 0.05. Factors abbreviations: Nu, Nutrients; Se, Seaweeds, Bold face values are significant at *P* < 0.05, *P*-values based on 999 permutations.*

**FIGURE 3:**
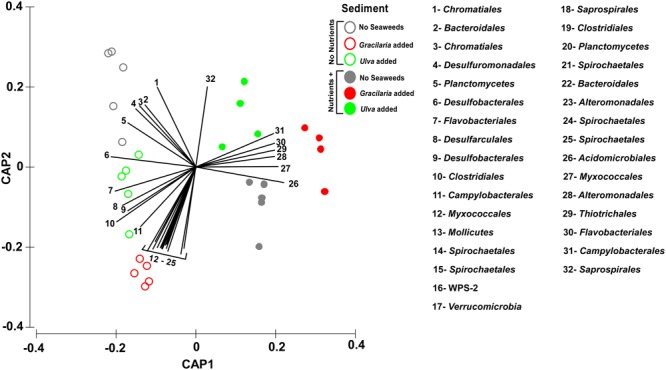
Canonical analysis of principal coordinates (CAP) ordination plot (based on Bray–Curtis of square-root transformed bacterial abundances) showing canonical axes that best discriminate the bacterial community assemblages across nutrient (open vs. closed circles) and seaweed addition (grey – no seaweeds, red – *Gracilaria*, green – *Ulva*). Order level OTUs with Pearson’s correlation >0.70 have been overlaid on the plot as vectors. Vector length corresponds to the strength of the correlation, with the circle representing the maximum correlation of 1. Image was manipulated by replacing the taxa names by numbers (with correspondence in lateral legend), in order to improve visualization. In addition, colors were changed so different treatment colors would match those of Figure 1.

At the OTU level, the bacterial community structure (abundance and composition) was affected by both seaweed and nutrient addition, independently of each other (Table 2A and Figure 3). For seaweed addition, *Gracilaria* strongly affected the structure of the bacterial community (SW0 vs. *Gracilaria p* = 0.024), whereas the Ulva addition had a marginal effect (SW0 vs. *Ulva p* = 0.079; *Gracilaria* vs. *Ulva p* = 0.405) (Table 2B). The *Gracilaria* addition (i) increased the average abundance of two *Desulfobacterales* order OTUs (denovo19603 and denovo36296) 20 and three times, respectively, (ii) increased the average abundance of a *Clostridiales* OTU by seven times (denovo1309), and (iii) doubled the average abundance of a *Campylobacterales* OTU (denovo60628). As such, increasing orders were involved in sulfate reduction and nitrogen fixation. In contrast, the average abundance of some specific OTUs of the order *Thiohalorhabdales* (denovo64034), *Chromatiales* (denovo6380), and *Marinicellaceae* (denovo60848) decreased 1.3 times with the addition of *Gracilaria* (SIMPER analysis, Supplementary Table S3A). *Gracilaria* addition also slightly increased the number of reads of methanogenic Archaea (*p* = 0.075), especially of Methanosarcinaceae and Methanomassiliicoccaceae members, and in that way affected Archaea composition (*p* = 0.015), whereas the nitrogen processing bacterial community was not driven by seaweed addition (*p* > 0.0359).

**Table 2A:** PERMANOVA main test for bacterial community β-diversity.

Source of variation	Pseudo-F	P (*perm*)	PERMDISP (P)
Nutrients	1.5954	**0.043**	0.99
Seaweeds	1.4574	**0.038**	0.12
Nu × Se	0.85528	0.808	0.06

*PERMANOVA values were calculated after square-root transformation and Bray-Curtis distances calculation for OTU composition. H_*0*_: there are no differences in the distribution of OTUs for the different treatments, H_*0*_ rejected if: *p* < *0.05*. Factors abbreviations: Nu, Nutrients; Se, Seaweeds, Bold face values are significant at *P* < 0.05, P-values based on 999 permutations.*

**Table 2B:** Results of PERMANOVA pair-wise test within the factor “Seaweeds” of OTU composition.

Groups	t	P (*perm*)
SW0, *Gracilaria*	1.3912	**0.024**
SW0, *Ulva*	1.2174	0.079
*Gracilaria, Ulva*	0.9895	0.405

*H_*0*_: there are no differences in the distribution of OTUs among seaweeds’ different treatments, H_*0*_ rejected if: *p* < *0.05*. Factors abbreviations: SW0, No Seaweeds, Bold face values are significant at *P* < 0.05, *P*-values based on 999 permutations.*

Although the nutrient addition did not affect the absolute N content and the C:N ratio of seagrass root fragments (*p* > 0.1600), it decreased the relative abundances of OTUs, predicted to be sulfate reducers (e.g., vectors 4, 5, 6, 8, 9, and 10 in Figure 3), whereas putative sulfur/sulfide oxidizers’ OTUs increased under nutrient loading conditions (vectors 29 and 31; Figure 3). The OTUs contributing most to this nutrient driven shift in community (recognized *S*-oxidizers and denitrifiers) increased when nutrients were added, with the most prominent being: *Campylobacterales* (denovo60628 and denovo42704) and *Chromatiales* (denovo64003), increasing 10, 17, and 9 times, respectively, (SIMPER analysis, Supplementary Table S3B). On the other hand, an OTU assigned to the sulfate reducer *Desulfobacterales* order (denovo19603) was reduced by 1.4 times in abundance by nutrient addition (SIMPER analysis, Supplementary Table S3B). The *Desulfobacterales*, also known as anaerobic N fixers, like *Clostridiales* and OTUs of both seaweed species, correlated negatively with the N addition (vectors 6, 9, 10, and 19 in Figure 3). For microaerophilic nitrogen-fixing *Campylobacterales*, two OTUs showed strongly contrasting correlations linked to nutrient addition (vectors 11 and 31 Figure 3) but coincided with *Ulva* addition.

#### Order Level Analyses

Considering only abundant orders (those in proportions higher than 1.5% and present in at least 60% of the replicates to assure representability of the entire replication), bacterial α-diversity did not differ among treatments, regardless of the indexes used (Supplementary Table S4 – Shannon diversity index and Chao I).

**FIGURE 4:**
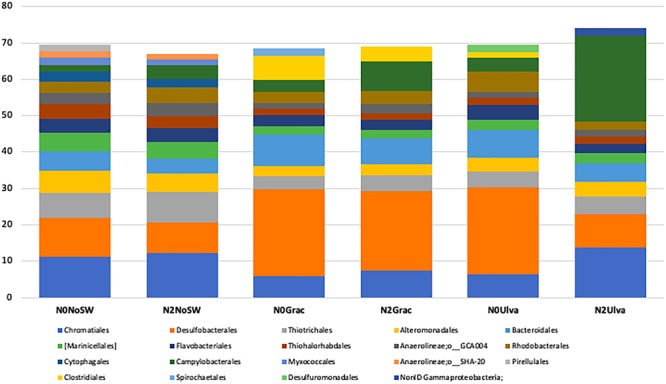
Mean bacterial community structure of sediments showing the most abundant (>1.5%) orders for each treatment, each represented by five replicate plots sampled, after filtering for the orders present in at least 60% of the replicates. Control – No seaweeds or nutrients added, N2SW0 – Only nutrients added, N0Grac – Only *Gracilaria* added, N2Grac – Both nutrients and *Gracilaria* added, N0Ulva – Only *Ulva* added, N2Ulva – Both nutrients and *Ulva* added.

For abundant orders, all plots without seaweed addition (irrespective of their nutrient treatment) displayed the same top six orders and equivalent proportions of putative sulfur/sulfide oxidizers (SO) and putative sulfate reducers (SR). Therefore, the bacterial community structure and composition were marginally affected by the nutrient addition (*p* = 0.056, Supplementary Table S5). Among the most abundant orders were *Chromatiales* (SO)- 11.2%, 12.3% (N2SW0); *Desulfobacterales* (SR)- 10.8%, 8.3% (N2SW0); *Thiotrichales* (SO)- 6.5%, 8.5% (N2SW0); and *Alteromonadales* (SR)- 5.5%, 5.1% (N2SW0) (Figure 4). Order level community structure/composition differed with seaweed treatment (*p* = 0.012, Supplementary Table S5A), with both seaweeds affecting sediment bacterial community equally (*Gracilaria p* = 0.008 and *Ulva p* = 0.016, Supplementary Table S5B). *Desulfobacterales* was the most abundant order in the seaweed addition treatments (N0Grac-23.9%, N2Grac-21.8%, and N0Ulva-23.8%), except in the N2Ulva treatment (9.2%) (Figure 4). Instead, *Campylobacterales* (SO) was conspicuously more abundant in the N2Ulva treatment (23.6%) compared with *Ulva* addition under ambient nutrient conditions (3.9%) and with the other treatments (Reference 1.9%, N2SW0 3.8%, N0Grac 3.4%, and N2Grac 8.2%) (Figure 4). The order *Bacteroidales* was the second most abundant only in seaweed-added sediments with ambient nutrient levels (8.5% for N0Grac and 7.7% for N0Ulva) but was also within the first orders for the combined nutrient and seaweed treatments (Figure 4). The order *Clostridiales* had an evident increase in *Gracilaria*-enriched sediments being very low to non-detectable in the other treatments (Reference- 0%, N2SW0- 0%, N0Grac-6.6%, N2Grac-4.2%, N0Ulva-1.5%, and N2Ulva-0%) (Figure 4). The heat map with samples clustering based on similarities given by the relative abundance of the main orders across all treatments shows a clear separation between the *Gracilaria* addition and the no seaweed addition treatments. Communities from plots where *Ulva* was added clustered either with the *Gracilaria* addition treatment (in the case of N0U), due to high abundances of *Desulfobacterales* (anaerobic N and S cyclers) or took an intermediate position between “no seaweeds” and the remaining “with seaweeds” treatments due to a unique high abundance of *Campylobacterales* (microaerophilic N fixers, Supplementary Figure S1; in the case of N2U). This position is more in agreement with OTU level beta-diversity multivariate analysis results that show an intermediate state of communities from the *Ulva* treatment, which is not statistically different from “no seaweeds” and *Gracilaria* treatments. Ambient plots without seaweed additions, as did the N2U treatment, showed distinct higher relative abundance of *Chromatiales* purpur sulfur bacteria known to contribute to P, S, and C cycling.

### Putative Functional Effects of Eutrophication and Algal Blooms

The PiCRUSt functional predictions had NSTI (Nearest Sequenced Taxon Index) scores ranging from 0.12 to 0.18, with an overall mean of 0.15 ± 0.02, a value even lower than that observed in the reference sediment. This indicated small average phylogenetic distances between the obtained 16S amplicon sequences and those of fully sequenced genomes used for PiCRUSt inferences suggesting likely accurate predictions. Predicted human-related functions/diseases were removed from the final table of functions (Supplementary Table S6).

The overall predicted functions, in contrast with the bacterial community structure, were only affected by seaweed and not by the nutrient addition (Table 3A), with both *Gracilaria* (*p* = 0.011) and *Ulva* (*p* = 0.016) similarly affecting the predicted sediment functional profile (Table 3B). Surprisingly, differences in predictive functionality with both seaweed additions were driven by increased cell motility and signal transduction and decreased Tryptophan and fatty acid metabolism and Valine, Leucine, and Isoleucine degradation (SIMPER).

**Table 3A:** Results of PERMANOVA main test for PiCRUSt functional predictions.

Source of variation	Pseudo-F	P (*perm*)	PERMDISP (P)
Nutrients	2.2561	0.117	
Seaweeds	3.4634	**0.016**	**0.007**
Nu × Se	0.75775	0.595	

*PERMANOVA values were calculated after square-root transformation and Bray–Curtis distances calculation for predicted functional profiles. H_*0*_: there are no differences in the distribution of predicted functions for the different treatments, H_*0*_ rejected if: *p* < *0.05*. Factors abbreviations: Nu, Nutrients; Se, Seaweeds, Bold face values are significant at *P* < 0.05, *P*-values based on 999 permutations.*

**Table 3B:** Results of PERMANOVA pair-wise test within the factor “Seaweeds” on predicted functional profiles.

Groups	t	P (*perm*)
SW0, *Gracilaria*	2.5101	**0.011**
SW0, *Ulva*	2.1480	**0.016**
*Gracilaria, Ulva*	0.9326	0.437

*H_*0*_: there are no differences in the distribution of OTUs among seaweeds’ different treatments, H_*0*_ rejected if: *p* < *0.05*. Factors abbreviations: SW0, No Seaweeds, Bold face values are significant at *P* < 0.05, *P*-values based on 999 permutations.*

**FIGURE 5:**
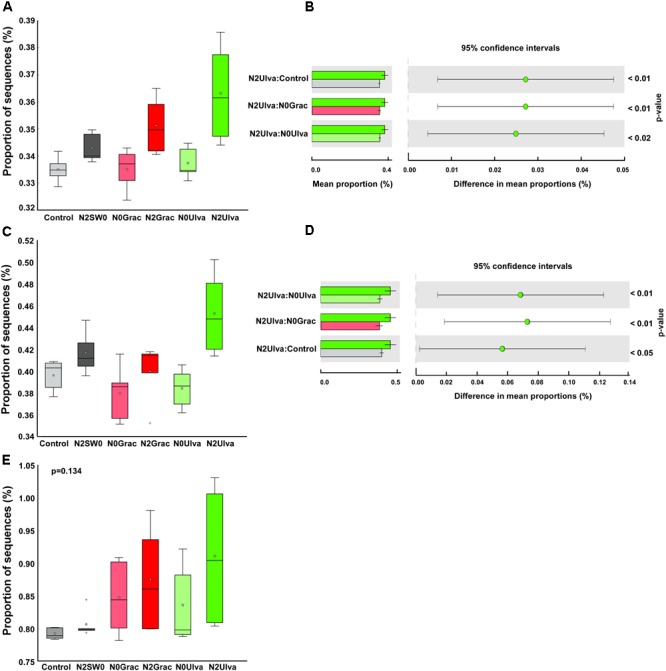
Box plot showing the distribution in the proportion of sequences assigned to the candidate functions: **(A)** Sulfur metabolism, **(C)** Sulfur relay system, and **(E)** Nitrogen metabolism. The top of the box indicates the third quartile, the bottom the first quartile, and the line in the middle is the median. Outliers are shown as crosses and the mean value as a star within the box. Right-side figures show Turkey–Kramer *post hoc* tests for each order where statistical differences were found **(B)** Sulfur metabolism, **(D)** Sulfur relay system, indicating the mean proportion of sequences within each treatment and the difference in mean proportions for each pair of treatment, with their corresponding pairwise *p*-value. Graphic colors were changed so different treatment colors would match those of Figure 1.

Although the overall functional predictions were only affected by the seaweed addition, the specific candidate KEGG categories related to the sulfur cycle were, in general, predicted to be more affected by the nutrient addition. Sulfur cycle-related sequences, such as the sulfur metabolism (*p* = 0.025) and the sulfur relay system (*p* = 0.004), were predicted to increase in sediments under the nutrient addition. This predicted increase was even more evident in the plots in which nutrients were combined with seaweeds, especially in the case of *Ulva* (Figures 5A–D). In contrast, nitrogen-cycling genes were predicted to be more, albeit insignificantly, affected by seaweed rather than the nutrient addition (Figure 5E), either linked to the increased variation in response among samples or reflecting the strong local effect of seaweeds.

**FIGURE 6:**
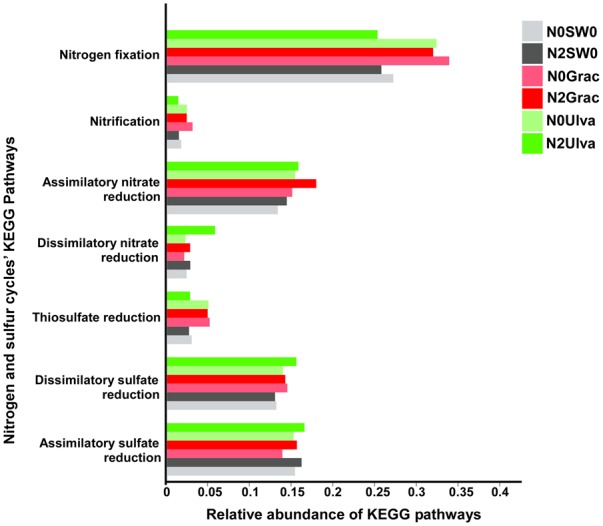
Relative abundance of nitrogen and sulfur cycles KEGG pathways for each treatment (with samples from the same treatment pooled together). Graphic colors were changed so different treatment colors would match those of Figure 1.

The KEGG genes specifically selected for nitrogen and sulfur cycles and pooled according to the different pathways (for samples pooled according to treatments), showed the presence of seven different pathways (Supplementary Table S7 and Figure 6). The nitrogen cycle pathways were: nitrogen fixation, nitrification, assimilatory nitrate reduction, and dissimilatory nitrate reduction – DNRA; and for the sulfur cycle: thiosulfate reduction, dissimilatory sulfate reduction, and assimilatory sulfate reduction. Statistically, the differences among treatments were not significant (*p* > 0.121; Supplementary Table S7), although some conspicuous predicted trends in some of the pathways for specific treatments are worth noting (Figure 6). Nitrogen fixation was predicted to show an elevated trend in seaweed treatments except for *Ulva* in combination with nutrients, in which the DNRA level seemed elevated (Figure 6). Concerning the sulfur cycle, both dissimilatory and assimilatory sulfate reduction were predicted to show similar relative abundances for all the treatments (Figure 6) and only thiosulfate reduction seemed to be predicted to display a higher relative abundance, of this pathway’s genes, for all the seaweed treatments except for *Ulva* combined with nutrients (Figure 6). Unfortunately, the PiCRUSt tool does not provide information on SOX genes (coding for thiosulfate oxidation – the main pathway in the sulfur cycle performed by sulfur-oxidizing bacteria), representing a large gap in the interpretation of the putative processes through our experiment.

At the order level, the closed reference PiCRUSt input table, used for functional inference, corresponded well with the table used for the bacterial community analysis. The readings associated with each of the most abundant orders were analyzed in PiCRUSt, targeting sulfur metabolism (the putative functional category of these orders). The treatment groups followed a similar trend (in terms of relative abundance) when comparing the most abundant orders with the proportion of sequences related to sulfur metabolism (Supplementary Figure S2). For example, *Campylobacterales* was only conspicuously abundant in the treatments where seaweeds (*Ulva*) and nutrients were combined (Figure 3 for OTUs, and Figure 4 for order level). The predicted proportion of sulfur metabolism related sequences of *Campylobacterales* was higher in nutrient addition and combined nutrient and seaweed treatments independently of the seaweed used (*p* = 0.007; Supplementary Figures S2A,B). Although some *Chromatiales* were strongly correlated with ambient sediments (vector 3 in Figure 3), the proportion of their predicted sulfur metabolism genes increased with the nutrient addition and they seemed to decrease with the seaweed addition under ambient nutrient conditions (*p* = 0.0001; Supplementary Figures S2C,D). For *Desulfobacterales*, the variation in the predicted proportion of sulfur metabolism genes increased with the addition of seaweeds (*p* = 0.254; Supplementary Figure S2E). As mentioned above, PiCRUSt did not provide information on SOX genes for the family function prediction.

For the carbon-related pathways found in the putative functional profile of PiCRUSt (Supplementary Table S6), we considered the different carbohydrate metabolism and carbon fixation pathways in prokaryotes (Supplementary Table S8). Similar to the nitrogen and sulfur cycles, no differentiation among treatments was detected (PERMANOVA *p* > 0.7553) with no conspicuous changes noted across treatments (Supplementary Figure S3). It is, however, worth noting that predicted carbon fixation by prokaryotes was the process contributing most to possible differences with an approximate 25% increase in the seaweed treatments, whereas these processes seemed to be decreased upon nutrient addition.

## Discussion

This study provides an important novel contribution to the current understanding of effects of combined nutrient and macroalgal loads on the variation of sediment bacterial communities, having identified the main taxonomical shifts and putative functional differences. Eutrophication episodes are associated with algal blooms, but so far, the cumulative (or not) effects of these two coupled ecosystem disruptors on sediment bacterial communities have barely been addressed. Here, for the first time to our knowledge, both nutrients and algal blooms (including the two natural blooming species) were experimentally manipulated in the field, in order to assess their individual and combined differentiation effects on bacterial communities in intertidal sediments. Our results clearly show that although eutrophication and algal blooms go hand in hand, they predominantly affected sediment bacterial communities differently and independently of one another. This will help us to better understand the impact of eutrophication in its different phases (nutrient increasing and consequent algal bloom) and the differentiated impact of different blooming algal species.

### Nutrient and Algae Shaping Sediment Bacterial Communities

The nutrient load affected the sediment bacterial community by decreasing diversity (alpha diversity) and changing its taxonomic composition (beta diversity), with the latter in agreement with previous studies (e.g., Campbell et al., 2010; Fierer et al., 2012; Guevara et al., 2014; Chen et al., 2015; Lawes et al., 2016; Liu et al., 2018). A low level of microbial diversity/richness is usually associated with an unhealthy and/or disturbed ecosystem (Allison and Martiny, 2008). Eutrophication through increasing nutrients is known to disrupt the local microbial structure through a cascade of processes that leads to unbalanced redox processes (Meyer-Reil and Köster, 2000). A pronounced shift in the microbial structure was evident in treatments where both seaweed and fertilizer were added.

Nutrient and seaweed addition affected the bacterial community composition in sediments independently. The seaweed effect was dependent on the seaweed species used, with *Gracilaria* causing a more pronounced community shift. *Ulva* loading resulted in a bacterial community that was not different from no seaweed and *Gracilaria* addition. However, *Ulva* in combination with the nutrient addition recorded a marked characteristic shift in the microbial structure.

Bacterial community composition in both sediment treatments, comparing only nutrient loading (without seaweeds), was rather similar, with the top six orders in close proportions and diversity remaining unchanged. This indicates that an increased amount of nutrients leads to bacterial community changes at a finer scale/lower phylogenetic level that is not reflected at higher taxonomic levels. Something similar was found by others authors (Koyama et al., 2014), which suggests the high plasticity of some taxonomic groups under a moderate disturbance. Kearns et al. (2016) also hypothesized that excess N would only favor a small number of taxa that are capable of responding to a nutrient increase.

However, where seaweeds were added, the differences were significant and evident even at the order level, as the addition of either of the seaweeds significantly changed the bacterial community similarly, irrespective of the seaweed species.

The bacterial community composition of the plots where *Ulva* was added along with nutrients was different when compared to the other seaweed-treated plots (including *Ulva* without nutrients). Together with the results at the OTU level, where the *Ulva* addition resulted in an “intermediate” community, this might reflect the different bloom strategies between these two seaweed species, as well as a fast turnover of *Ulva* biomass. These two fast-growing seaweeds have different blooming strategies, but both are recognized to profit from global eutrophication and its collateral effects as, for example, ocean acidification (Valiela et al., 1997; Young and Gobler, 2016). However, although *Gracilaria* can also benefit from nutrient increasing by increased growing rates (Ye et al., 2013), *Ulva* grows faster with high nutrient concentrations (Wallace and Gobler, 2014; Young and Gobler, 2016). Moreover, there are structural differences, where *Gracilaria* presents a much more robust conformation. These differences and potential repercussion in bacterial communities of the sediments will be discussed further.

### Main Bacterial Taxa Shifting Across Treatments

Overall, the results showed that the most abundant taxa in all treatments were related to *Proteobacteria* and to sulfate-reducing and sulfur/sulfide-oxidizing (*S*-oxidizers) bacteria. This suggests, as predicted, an active sulfur cycle under the sediment conditions of this ecosystem. The vectors plotted and separating the two main “nutrient vs. non-nutrient” clusters suggest an increase of sulfur oxidizers when nutrients are added (Figure 3). This tendency was also followed by the OTUs that contributed most to this difference in similarity percentages analysis. OTUs affiliated to the orders *Campylobacterales* (denitrifiers) and *Chromatiales* (both recognized as *S*-oxidizers) increased in nutrient-loaded sediments (especially those combined with seaweeds). In coastal sediments affected by anoxic and sulfidic conditions, DNRA can be the predominant nitrate reduction pathway (Brunet and Garcia-Gil, 1996; Christensen et al., 2000; McGlathery et al., 2007) in which *S*-oxidizing bacteria play a crucial role (Christensen et al., 2000; An and Gardner, 2002; Song et al., 2014). DNRA might be favored over denitrification at high organic C loading (Hardison et al., 2015), such as might be the case during joint nutrient and seaweed loading (however, denitrification was using PiCrust not found in our data).

At the order level, nutrient loading alone was not reflected in a changing bacterial community. Two of the most abundant orders, *Chromatiales* and *Thiotrichales* (most of them affiliated to *Thiotrichaceae* family), were also the two orders that slightly increased in abundance when nutrients were added (Figure 3). These two orders are known dominant sulfur oxidizers in salt marsh sediments (Thomas et al., 2014) and are recognized for their potential roles in coupled N and S cycles (Ganesh et al., 2015), which could grant them an advantage when nutrient concentrations increase.

Except for the combination of *Ulva* and nutrients, all the sediments loaded with seaweeds (regardless of the species) showed a high increase of bacteria belonging to the order *Desulfobacterales*. These, predominant in seaweed-enriched sediments (Morrison et al., 2017), are sulfate-reducers that oxidize organic compounds and produce hydrogen sulfide as the metabolic end product (Lamers et al., 2013). During algal blooms, the organic matter deposited in the sediments results in intense microbial activity due to the high decomposition rates (Fenchel and Jørgensen, 1977). The upper layer of sediments will become anoxic along with the release of toxic sulfide resulting from the metabolism of these abundant sulfate reducing bacteria. The addition of algal organic matter to the sediment may have become advantageous to this strictly anaerobic, metabolically diverse group of bacteria. These groups also have larger genomes relative to other sulfate-reducing bacteria, which allow a faster response to stronger environmental perturbations and are stimulated by the release of carbon compounds (Kearns et al., 2016).

A puzzling effect was the different response of the nutrient and *Ulva* combination plots, in which there was an increase in the abundance of *Campylobacterales* order instead of *Desulfobacterales*. The order *Campylobacterales* is the second most abundant in treatments with *Gracilaria* and nutrients combined, although the difference is three times higher for *Ulva*. As *Campylobacterales* (*Epsilonproteobacteria*) are hardly found on seaweeds (Hollants et al., 2012), it is highly unlikely that their presence in these treatments comes from the seaweeds. In addition, bacterial algal cell wall degraders are from other groups, mainly *Alpha*- and *Gammaproteobacteria* and members of the CFB group possessing specific sugar-degrading enzymes such as agarases, carrageenases, and alginases and for *Ulva* specifically ulvan lyase (Hollants et al., 2012). As mentioned above, these seaweeds have different blooming behaviors and tissue conformation. Both take advantage of high nutrient concentrations, but *Ulva* grows faster than *Gracilaria* (Young and Gobler, 2016). This difference in growth rates, allied with a less robust conformation in *Ulva*, may result in asynchronous decomposition rates and timings, which can be mirrored in the bacterial community of the sediments sampled in the same time frame for all the treatments. Also, due to its higher uptake capacity, *Ulva* holds a high nutrient content (C:N ratio), usually reflecting environmental increments (Sjöö and Mörk, 2009), which causes it to decompose more rapidly (Teichberg et al., 2010) than *Gracilaria* (Enriquez et al., 1993). The taxonomic differences found among *Gracilaria* and *Ulva* plots do not reflect the differences found between the associated communities of these two seaweeds (Lachnit et al., 2011). Observations during the experiment showed that *Ulva* was the fastest-disappearing seaweed from the plots. *Campylobacterales* (here mostly affiliated to the genus *Sulfurimonas*, see Supplementary Table S1) are mostly sulfide oxidizers commonly found in pelagic marine redox-clines (Campbell et al., 2006). Following *Ulva*’s faster decomposition and the putative high production of sulfides during the process, the bloom would collapse allowing some oxygenation of the sediments. Accounting for a large percentage of denitrification genes, *Campylobacterales* may also be responsible for the removal of the nitrates, through their use for sulfide oxidation (Llorens-Marès et al., 2015). Liu et al. (2011) analyzed the bacterial communities in the seawater during *Ulva* blooms and detected *Campylobacterales* as the dominant bacterial clade when the bloom was collapsing and the algal material was decaying. This suggests that this group of bacteria might be taking advantage of the high concentration of nutrients and organic matter in decomposition.

The order *Bacteroidales* also increased in abundance in the plots where seaweeds were added independently of their nutrient status. Members of this order include efficient decomposers/digesters of dead organic matter, which are usually found in sludge, contributing to the degradation of proteins (Rivière et al., 2009) and have been found in seaweed enriched sediments (Morrison et al., 2017). Coupled with their decomposition skills, *Bacteroidales*, are also able to couple the electron flow from organic matter to reduce nitrate to ammonium (Llorens-Marès et al., 2015).

It is also important to mention that the order *Clostridiales* was among the most abundant orders for *Gracilaria* enriched treatments and was also present (although much less abundant) in no-nutrient *Ulva* enriched sediments. *Clostridiales* are strict anaerobes, known to mediate the degradation of complex carbohydrate polymers with the capacity for cellulose degradation (Vanwonterghem et al., 2014). They have been found associated with fermentation of recalcitrant plant cell wall polysaccharides and have been found in environments with high plant biomass turnover rates (van der Lelie et al., 2012). The elevated presence of this order in seaweed-enriched sediments, with a notably higher proportion in *Gracilaria* plots, is in line with the high amount of organic matter in these plots, and the harder tissue structure and the stronger burial survival of *Gracilaria* compared to *Ulva*. *Gracilaria* is, in general, also (partly) buried in the mud in the Ria Formosa, which might actually stimulate anaerobic conditions required for *Clostridiales* members. *G. vermiculophylla* is highly resistant to burial, desiccation, and grazing and growth is not very dependent on nutrient levels (Thomsen and McGlathery, 2007), which might explain the similar behavior of bacterial communities under both nutrient treatments within the *Gracilaria* addition and the strong differences with *Ulva* enrichment treatments. The fact that this order was not detected in the treatment where *Ulva* was added along with nutrients, is consistent with the hypothesis of an already advanced decomposition in these plots. In future experiments, physicochemical and geochemical parameters should be monitored continuously (e.g., oxygen levels, C:N ratios, decomposition rates, organic matter content) to assess possible relationships with the bacterial community guilds.

### Predicted Functional Effects of Eutrophication and Algal Blooms

PiCRUSt can be used as an exploratory tool to help to decide on a more expensive high-depth metagenomic sequencing approach, due to the demonstrated consistency in predicting functional diversity based on phylogenetic affiliation (e.g., Staley et al., 2014), with good results demonstrated for soil and sediments (Langille et al., 2013). However, being aware of these prediction limitations and possible flaws, as well as the controversy around the use of this tool, this discussion will be general and limited to raising pertinent hypotheses. Therefore, we only focused on candidate pathways from the sulfur cycle, coupled with the nitrogen cycle, which is one of the most important biogeochemical cycles in coastal sediments (Rysgaard et al., 1996).

Most of the work done so far suggests that, as a result of disturbance, sediment bacterial communities change their functional profile along with community structure (as reviewed by Allison and Martiny, 2008). Most recently it has been found that, even when bacterial composition remains unaltered through community resilience, the functional capacity is adapted by a shift in the portion of actually active bacteria (Kearns et al., 2016).

Statistical differences found for predicted metagenomes are more comparable with those achieved with the main order levels than with the OTU level. The overall (all the pathways considered) functional profiles predictions are distinct for seaweed-enriched sediments but do not differ between treatments with the seaweed species. However, significance in seaweed treatments may reflect functional instability among treatments, related to the different decomposition timings discussed above. Functional profiles of chosen candidate pathways show that the number of reading assigned to the sulfur cycle increased in the nutrient treatments and, more sharply, when seaweeds are combined. Sulfur cycle stimulation has been reported in coastal lagoons, particularly sulfate reduction rates in anoxic conditions caused by organic enrichments (Holmer et al., 2004; Duarte et al., 2005). Seaweed-enriched sediments can have an upregulation of *dsr* genes, which encode proteins responsible for dissimilatory sulfate reduction (Morrison et al., 2017). The readings assigned to the nitrogen cycle followed a similar pattern as those of the sulfur cycle. Seaweed addition is likely the determinant factor and its effect is enhanced when combined with nutrients. These two cycles are coupled and their dynamics allows the efficiency of this ecosystem service. However, as the amount of sulfide can be six times higher than that of ammonium in coastal lagoons, the abundance of sulfur-related genes is expected to be higher (Laverock et al., 2011), in agreement with our results. Sulfate reduction is considered the most important anaerobic respiratory process occurring in coastal lagoons (Caumette et al., 1996).

Zooming into sulfur and nitrogen cycles specific pathways corroborates some of the hypotheses advanced for the bacterial community taxonomical shifts. The relative abundances of reads assigned to DNRA were higher in the treatment where *Ulva* was combined with nutrients. The lack of SOX genes in the PiCRUSt database did not allow us to infer sulfur oxidation activity, although it is known that sulfur oxidation is one of the pathways used by some DNRA species (Burgin and Hamilton, 2007). The higher abundance of the sulfur-oxidizers/denitrifiers – *Campylobacterales-* in this treatment, probably reflects the increase of these processes in the N2Ulva treatment. In contrast, nitrogen-fixation is higher in all seaweed treatments except for N2Ulva. An experiment in nitrogen-rich coastal surface waters demonstrated a higher abundance of *nifH* genes in surface waters covered with massive macroalgal canopies (Zhang et al., 2015). Surprisingly, the relative abundance of reads putatively assigned to sulfate-reduction (dissimilatory and assimilatory) was equivalent for all treatments. However, those putatively assigned to thiosulfate-reduction (also performed by sulfate-reducing bacteria) seemed higher in seaweed-treated sediments (except N2Ulva), which is in line with the higher abundance of sulfate-reducing bacteria (*Desulfobacterales*) in these treatments.

In the Carbon cycling category, the relative abundance of sequences associated with carbon-fixation pathways in prokaryotes slightly increased in the seaweed-treated plots. That might be related to the seaweed degradation in the seaweed-added plots and the consequent fixation of the released CO2 by sediment bacterial community. This study constitutes a scaffold to be considered in further analyses targeting specific genomic and/or transcriptomic pathways that are required to validate our hypothesis. These analyses, paired with physical/geochemical data, would allow a more complete picture of the taxonomical and functional shifting in sediments under coupled environmental stress.

### Caveats and Considerations

In this experimental field study, nutrient and seaweed levels were not kept constant throughout the experimental duration. The use of slow-release fertilizer and a pulse loading of seaweeds aimed to simulate natural field conditions of this lagoon. Seaweed blooms on intertidal banks are variable both in time and space due to the ecological processes, but also due to a to lack of hard substrate for fixation and to variable tidal currents. After 5 weeks at the point of sampling, most seaweed biomass was lost from the experimental plots and, as such, our results mostly represent the post-bloom conditions. Although effects of nutrient and seaweed addition were experimentally tested, we did not assess the temporal changes in bacterial communities, which likely changed during the duration of the experiment. In addition, the effects documented are a combination of indistinguishable direct and indirect treatment effects on the entire ecosystem present. Both treatments may not only affect the bacterial communities directly but probably also affect eukaryotic fauna in and on sediments. Infauna activity, for example, directly affects the oxygenations of sediments by bioturbation, biogeochemical cycling, as well as the processing of seaweed biomass, and this has consequences for the bacterial community structure and function in sediments (Herbert, 1999). However, the objective was not to identify the mechanisms, but rather to assess the net effects of nutrient and seaweed addition on bacterial communities in the field. Despite all this natural variability that takes place in field conditions, the experimental design ensures that the differences between treatments can be attributed to the factors tested, and not to other sources of variability that were affecting all plots equally.

Although we are very cautious in the use of PICRUSt for providing functional insights from our nonhuman model systems, the low NSTI values and the good match between the main taxonomical and predictive functional differences provide confidence in the used approach in this study. Gene expression data would have provided more accurate functional insights but would probably also be more specific for the time and conditions of sampling.

Environmental metadata could have provided us with clues regarding the specific environmental changes that our treatments would have caused, concerning nutrient conditions for example. Rather than multiple samplings across the duration of the experiments to adjust for strong spatial variability in intertidal environments, we opted for a more time-integrated approach using the C:N ratios of macrophyte biomass in each plot. Unfortunately, the obtained biomass showed no differences among treatments, despite documented internal N pool and environmental dissolved inorganic nitrogen concentrations (Teichberg et al., 2010). We believe this is more a consequence of the used method, partly due to the turnover rate of biomass during the experimental 5 weeks than the treatment considering the strong effects on the bacterial community. Redox, pH, oxygen, and sulfide electrode profiles would have been extremely valuable to correlate with community changes.

Eutrophication and macroalgal blooms are challenging for coastal marine management. Although both lead to elevated microbial activity resulting in anoxia with ecosystem and social implications, this study demonstrated that nutrient and seaweed enrichment drive microbial communities in sediment into different compositions and structure. Algal blooms could be considered to be nature’s solution for buffering short term eutrophication by transferring inorganic into organic and subsequently “slowly” recycling these compounds into the system by different microbial communities specialized in degrading complex compounds. Overall, however, it seems that the natural solution in the form of macroalgal blooms has a larger impact on sediment microbial communities than nutrient loading does. For coastal management, our findings show that reducing eutrophication sources will be most effective. Macroalgae can be effectively used for nutrient capturing, but they should be harvested in time. In addition, introduced *Gracilaria* seems to affect sediment microbial community composition and functioning, as well as biogeochemical cycling, differently from blooms of native *Ulva*. Sediment microbial communities could provide considerable information as indicators of ecosystem status, similarly to macro-organism indices.

## Conclusion

Both nutrient and seaweed additions to sediments have the net capacity to change (directly or indirectly) the microbial structure of coastal sediments and act mostly independently of one another. Seaweed addition effects were more pronounced than nutrient addition effects and were species-dependent. The most important bacterial community changes (regarding taxa abundance) occurred among bacteria involved in nitrogen and carbon, but especially in sulfur cycling.

## Author Contributions

AE designed the study and was responsible for the field experiment execution and final sampling. TA extracted DNA, amplified 16S rRNA genes and performed all *in silico* analysis. TA, GM, ES, and AE drafted the manuscript. All authors approved the final manuscript.

## Conflict of Interest Statement

The authors declare that the research was conducted in the absence of any commercial or financial relationships that could be construed as a potential conflict of interest. The reviewer LK and handling Editor declared their shared affiliation.

## Footnotes

1 www.researchandtesting.com

2 http://www.mothur.org/w/images/0/0c/Wet-lab_MiSeq_SOP.pdf

3 https://huttenhower.sph.harvard.edu/galaxy

4 http://metagenomics.anl.gov/linkin.cgi?project=mgp21934
