# Supplementary material for: Seaweed Loads Cause Stronger Bacterial Community Shifts in Coastal Lagoon Sediments Than Nutrient Loads
Source: Front Microbiol. 2019 Jan 9;9:3283. doi: 10.3389/fmicb.2018.03283 (PMC6333863; doi:10.3389/fmicb.2018.03283)
Supplement: Supplementary file 9 [file Image_1.PDF]

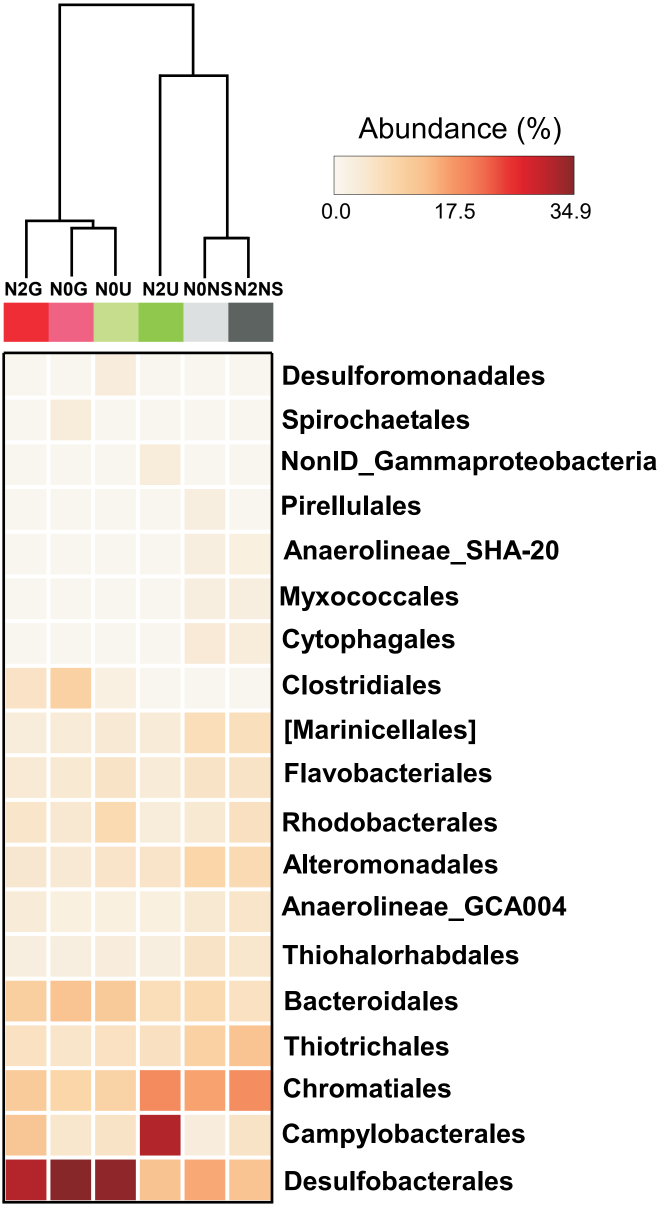

Fig.S1. Heat map clustering the different treatments independently using Ward clustering method based on the main orders' (used in Fig.3) relative abundances. The data matrix's rows and columns were permuted according to corresponding clustering, bringing similar treatments closer to each other. Ward's clustering method (with a 0.75 threshold) was used in the main orders' relative abundances (the same data as in Fig.4). Colors were changed so different treatment colors would match those of Fig.1.
