# Supplementary figures and images for: Seaweed Loads Cause Stronger Bacterial Community Shifts in Coastal Lagoon Sediments Than Nutrient Loads
Source: Front Microbiol. 2019 Jan 9;9:3283. doi: 10.3389/fmicb.2018.03283 (PMC6333863; doi:10.3389/fmicb.2018.03283)

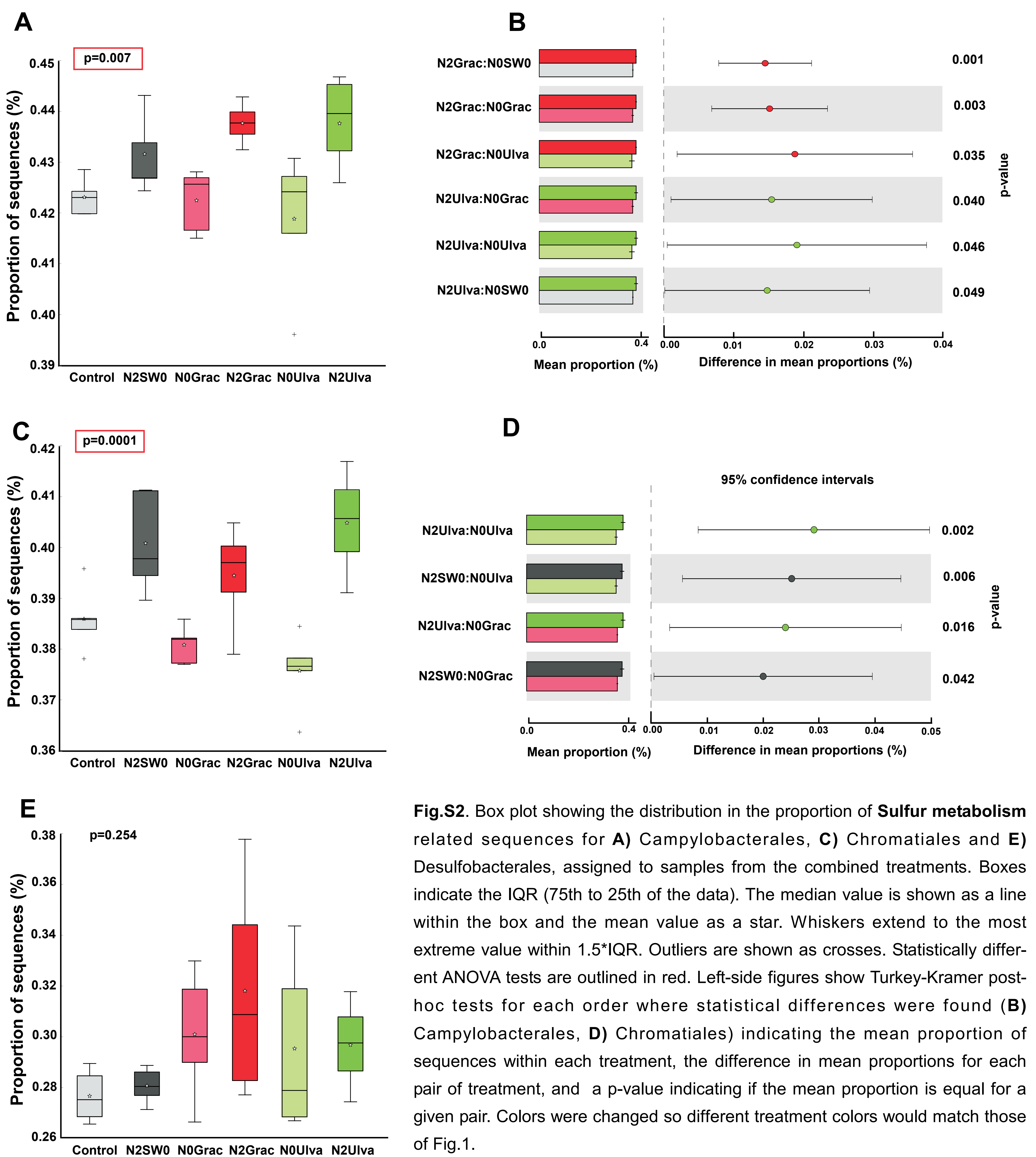

Supplement: Supplementary file 10 [file Image_2.PDF]

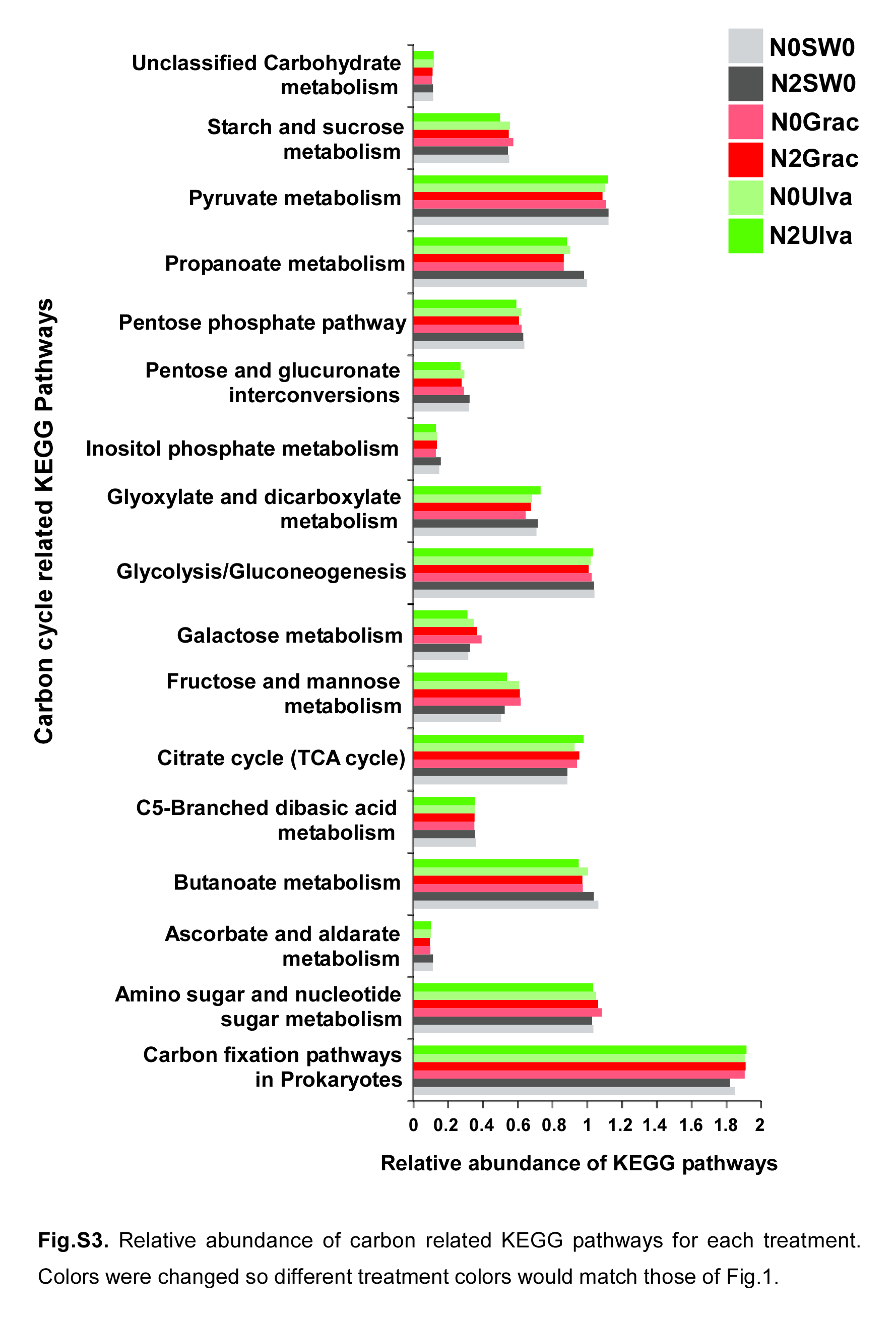

Supplement: Supplementary file 11 [file Image_3.TIF]
